# Intermittent cold stimulation acclimates broilers to acute cold stress by affecting cardiac lipid metabolism

**DOI:** 10.5713/ab.24.0389

**Published:** 2024-10-25

**Authors:** Yong Zhang, Minghang Chang, Qiang Xue, Hongyu Wang, Yuanyuan Liu, Haidong Wei, Jianhong Li

**Affiliations:** 1 College of Life Science, Northeast Agricultural University, Harbin 150030, China; 2 College of Animal Science and Technology, Northeast Agricultural University, Harbin 150030, China; 3 Key Laboratory of Chicken Genetics and Breeding, Ministry of Agriculture and Rural Affairs, Harbin 150030, China

**Keywords:** Broiler, Heart, Intermittent Cold Stimulation, Lipid Metabolism

## Abstract

**Objective:**

This study aimed to investigate whether intermittent cold stimulation can induce adaptation in broilers to acute cold stress (ACS) by regulating the lipid metabolism of hearts.

**Methods:**

CS0 were kept at normal rearing temperature, while CS3 and CS5 were exposed to 3°C for 3 and 5 hours, respectively, on alternate days lower than CS0 from 15d to 35d. On 50d, broilers in three groups were exposed to ACS at 10°C for 12 hours (Y12). The levels of corticosterone (CORT) and liothyronine (T3), mRNA and protein levels of heart adenosine monophosphate (AMP)-activated protein kinase/mammalian target of rapamycin (AMPK/mTOR) pathway genes were assessed at 36 d, 50 d and Y12.

**Results:**

At 36d, mRNA levels of AMPKα, acyl-CoA oxidase (ACO), mTOR, sterol-regulatory element binding protein (SREBP), stearoyl-coA desaturase (SCD), acetyl-coA carboxylase (ACC), fatty acid synthase (FAS) and protein level of peroxisome proliferators-activated receptor α (PPARα) in CS3 and CS5 were significantly lower than those in CS0 (p<0.05). At 50d, compared to CS0, mRNA levels of PPARα, carnitine palmitoyltransferase1 (CPT1), ACO, tuberous sclerosis complex (TSC), SREBP and SCD, as well as protein levels of p-AMPKα/AMPKα, PPARα and SREBP were significantly increased in CS5 (p<0.05). At Y12, the levels of T3 in CS3 and CS5 were significantly higher than those in CS0 (p<0.05), mRNA levels of CPT1, ACO, SREBP, SCD and protein levels of p-AMPKα/AMPKα, SREBP, and FAS were significantly higher in CS5 than in CS0 and CS3 (p<0.05). However, compared to 50d, at Y12, mRNA levels of AMPKα, CPT1 and ACO in CS3 and CS5 significantly decreased (p<0.05), while protein levels of p-AMPKα/AMPKα significantly increased (p<0.05).

**Conclusion:**

This study suggested that intermittent cold stimulation at 3°C lower than normal rearing temperature for 5h could help broilers adapt to the ACS by promoting heart lipid metabolism.

## INTRODUCTION

Rapidly growing broilers are susceptible to various environmental stressors, including cold stress, which is one of the limiting factors for broiler production in northern China. Previous studies have indicated that the increase in cardiovascular disease mortality is positively correlated with a decrease in environmental temperature [1]. Cold stress can also lead to heart hypertrophy, interstitial fibrosis and reduced heart ejection fraction [2]. It is worth noting that exposure to cold stress cause neuroendocrine disruption and increased heat production, accompanied by the generation of heart oxidative stress [3]. Additionally, cold stress has been reported to impair heart energy status by reducing heart energy reserves and fatty acid oxidation capacity in broilers [4].

The ability of broilers to respond to cold stress depends on the appropriate involvement of the central and peripheral nervous systems, coordinating hormonal and immune regulation among multiple organ systems to establish homeostasis [5]. The hypothalamic-pituitary-adrenal (HPA) axis and hypothalamic-pituitary-thyroid (HPT) axis are integral components of the neuroendocrine system, secreting hormones such as corticosterone (CORT) and liothyronine (T3) to regulate responses to cold stress, including energy balance, thermoregulation and cold-induced thermogenesis [6]. CORT serves as the primary effector hormone of the HPA axis, reflecting the level of HPA axis activation under cold stress and influencing nearly all physiological systems, thereby redistributing energy or nutrient resources within biological systems. For instance, previous research has shown that cold stress significantly increases CORT levels in the blood of piglets and mice, accompanied by elevated levels of glucose and lipid substances, aiding in their adaptation to cold stress [7]. Cold stress also activates the HPT axis, subsequently leading to elevated levels of T3 and thyroxine (T4) in the blood [8]. Xie's study [9] found that cold stress accelerates the conversion of T4 to T3 in laying hens, thereby increasing blood T3 level to enhance catabolic metabolism, increase heat production, and promote the synthesis of intracellular proteins, RNA and DNA, thereby resisting cold stress damage. Therefore, investigating the changes in neuroendocrine hormones in broilers under cold stress is of significant importance for studying their physiological responses to cold stress and metabolic changes.

The adenosine monophosphate (AMP)-activated protein kinase/mammalian target of rapamycin (AMPK/mTOR) pathway is essential for restoring internal balance following stress stimulation. It is generally believed that when there is a relative increase in intracellular AMP or adenosine diphosphate (ADP) levels, the AMPK pathway stimulates downstream genes, such as peroxisome proliferators-activated receptor α (PPARα), carnitine palmitoyltransferase1 (CPT1) and acyl-CoA oxidase (ACO), thereby inhibiting synthetic metabolic pathways and promoting catabolic pathways to replenish adenosine triphosphate (ATP) supply in broilers [10]. Zhou et al found that cold stress induces enhanced expression of the AMPK gene in the intestines of broilers and upregulates the expression of intestinal tight junction genes, demonstrating a potential link between cold stress, AMPK and intestinal barrier function [11]. Study also has found that the expression levels of AMPKα, PPARα and CPT1 in broiler muscle were increased under nutrient deficiency conditions, which was thought to be related to the increased level of fatty acid oxidation [12]. As important downstream genes and regulatory nodes of the AMPK pathway, mTOR and its target genes exert effects that are precisely opposite to those of the AMPK pathway. Li et al [4] reported that in mice fed high glucose, the liver AMPK pathway was inhibited, while sterol-regulatory element binding protein (SREBP) and its downstream stearoyl-coA desaturase (SCD) and acetyl-coA carboxylase (ACC) were activated, resulting in non-alcoholic fatty liver in the mice. RNA sequencing results on chicken DF-1 cells revealed downregulation of genes including oxidative phosphorylation, ribosomes and mTOR after low-temperature stimulation. These genes primarily impact ribosomal translation and mitochondrial electron transport in DF-1 cells [13]. Although the role of the AMPK/mTOR pathway in skeletal muscle and liver has been well studied, its role in other metabolically active organs (such as the heart) remains largely unknown.

The detrimental effects of cold stress on heart function have been widely acknowledged, but research on how to alleviate cold-induced heart injury is relatively scarce. However, our previous studies have demonstrated that appropriate intermittent cold stimulation can significantly improve broiler performance and immune function, effectively mitigating acute cold stress (ACS) injury [14–16]. Therefore, we hypothesized that appropriate intermittent cold stimulation could regulate the AMPK/mTOR pathway by affecting neuroendocrine hormone secretion, thereby regulating heart lipid metabolism and enhancing cold adaptability of broilers.

## MATERIALS AND METHODS

### Animals and experimental design

All experimental procedures performed in this study were approved by the Animal Care and Use Committee of Northeast Agricultural University (IACUCNEAU20150616).

A total of 240 healthy 1 d Ross 308 broilers were housed in three artificial climate houses, and the immunization procedures of all broilers were strictly performed according to broiler production standards. At 15 d, they were randomly divided into control group (CS0 group), 3h cold stimulation group (CS3 group) and 5 h cold stimulation group (CS5 group). Each group had five replicates with 16 broilers per replicate.

**Figure 1:**
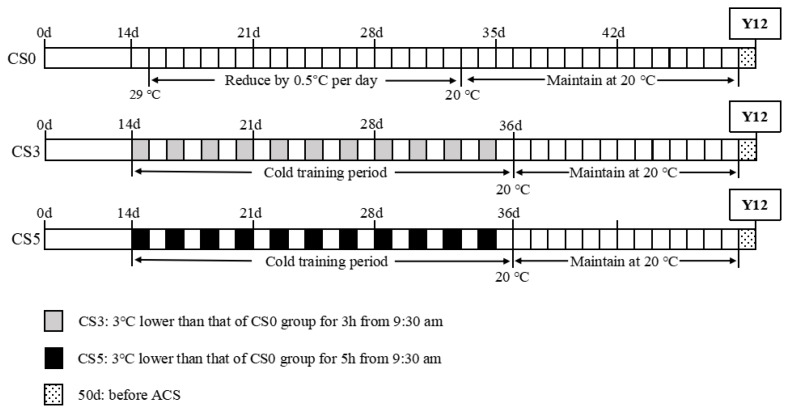
The specific rearing temperature. ACS, acute cold stress; Y12, ACS at 10°C for 12h from 9:30 am.

All broilers were initially housed at the constant temperature from 1 to 14 d, maintained at 35°C from 1 to 3d, and then gradually decreased by 1°C every 2 days. From 15 to 49 d, CS0 group remained at the normal rearing temperature. This temperature was reduced by 0.5°C per day until reaching 20°C on 33 d, and it was maintained at this temperature until 49 d. However, CS3 and CS5 groups were exposed to intermittent cold stimulation at a temperature of 3°C lower than that of CS0 group from 9:30 am on the 15 d. The duration of cold stimulation was 3 hours for the CS3 group and 5 hours for the CS5 group, occurring once every two days until 35 d. From 36 d to 49 d, the temperature was maintained at 20°C in all three groups. At 50 d, all three groups were subjected to ACS for 12 hours (Y12), with the rearing temperature reduced to 10°C at 9:30 am. The specific rearing temperature was shown in Figure 1.

### Sample collection

One broiler from each group per replicate was randomly selected (n = 5 each group) and sacrificed by neck dissection at 36 d, 50 d and after ACS for 12h (Y12). Blood samples were collected in 10 mL centrifuge tubes and centrifuged at 1,500×g for 10 min at 4°C to separate serum for subsequent Enzyme-Linked Immunosorbent Assay (ELISA) tests. After dissection, the hearts of broiler were quickly collected in EP tubes, snap frozen in liquid nitrogen, and then frozen at −80°C for subsequent quantitative polymerase chain reaction (qPCR) detection and Western blot experiments.

### Enzyme-linked immunosorbent assay

Serum T3 and CORT levels in each group at 36 d, 50 d and Y12 were measured using ELISA kits (Xinle Biotechnology; Shanghai, China) according to the manufacturer’s instructions. The absorbance (OD) of each serum sample at 450 nm was measured using a microplate reader (Roche, Basel, Switzerland), and the levels of T3 and CORT were calculated.

### RNA extraction and gene expression assays

Total RNA was extracted from broiler heart samples using Trizol (Takara, Kusatsu, Japan) following the manufacturer’s instructions. The extracted RNA was reverse transcribed to synthesize cDNA using ReverTra Ace qPCR RT Master Mix and gDNA Remover (Toyobo, Osaka, Japan) as per the provided instructions, and stored at −80°C.

The primers were designed with Primer Premier 5.0 (Premier Biosoft International, Palo Alto, CA, USA) and synthesized by Biotechnology company (Sangon, Shanghai, China). The primer sequences of the related genes were shown in Table 1.

The qPCR amplification was performed using a Light Cycler 96 qPCR system (Roche). The reaction used a 10 μL mixture, including 5 μL of the Roche Fast Universal SYBR Green Master kit (Toyobo), 1 μL of cDNA sample, 0.3 μL of forward and reverse primers, and 3.4 μL of DEPC water [16]. The relative mRNA expression levels of the target genes were calculated by the 2 −ΔΔCT method, with the β-actin gene as an internal reference.

### Tissue protein extraction and western blot analysis

Total protein was extracted from frozen broiler heart tissue samples using Western IP cell lysate (SparkJade, Harbin, China) with 1% phenyl methane sulfonylfluoride (PMSF) (SparkJade, Shandong, China). Protein concentrations were quantified using the BCA Protein Assay kit (SparkJade) and adjusted to the same concentration (4 μg/μL). The same volume (10 μL) of total protein was plated onto a 10% gel (SparkJade) for sodium dodecyl sulfate polyacrylamide gel electrophoresis (SDS-PAGE). Proteins were transferred to nitrocellulose membranes (SparkJade) using a semi-dry transfer device (Amersham Biosciences, Boston, MA, USA). After blocking the nitrocellulose membrane with 5% skim milk for 2 h at 37°C, the membrane was incubated with specific primary antibodies at 4°C overnight, and the diluted ratio of primary antibodies were shown in Table 2. Following primary antibody incubation, second antibody IgG-HRP was incubated (1:9,000; ZEN-BIOSCIENCE, Chengdu, China). The protein bands were observed on a gray-scale scanner (Gene Gnome XRQ, Cambridge, UK) using the ECL chemiluminescence kit (SparkJade). Finally, Image J (NIH, Bethesda, MD, USA) was employed to analyze the gray scale of the bands, and the ratio of the gray value of each target protein to the gray value of β-actin protein was calculated to represent the relative expression of the target protein.

### Statistics analysis

Data were analyzed using IBM SPSS Statistics 21.0 (IBM, Armonk, NY, USA). After assessment of a normal data distribution with the Kolmogorov-Smirnov test, One-way ANOVA was used to analyze the difference and Duncan's test was used for multiple comparisons. All results were expressed as "mean±standard error of means". A p-value of <0.05 was considered significant.

## RESULTS

### Effects of intermittent cold stimulation and acute cold stress on neuroendocrine hormones levels

**Figure 2:**
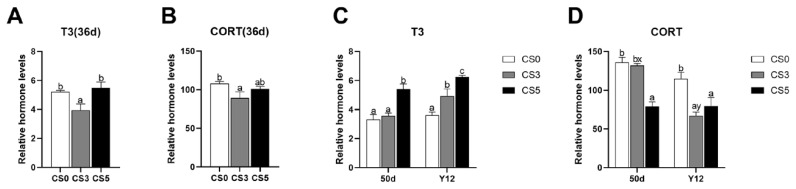
Effects of intermittent cold stimulation and ACS on neuroendocrine hormones levels in broilers. (A), (B) Effect of intermittent cold stimulation, (C), (D) effect of ACS. T3, liothyronine; CORT, corticosterone; ACS, acute cold stress. ^a–c^ The difference between the treatment groups was statistically significant (p<0.05). ^x,y^ Represent the difference between the same treatment group at 50–d and Y12 (p<0.05).

The effects of intermittent cold stimulation on neuroendocrine hormones levels are shown in Figures 2A, 2B. At 36 d, CS0 group and CS5 group had significantly higher levels of T3 hormone than CS3 group (p<0.05). CS0 had a significant higher level of CORT than CS3 group (p<0.05). However, there were no significant differences in CORT level between CS0 group and CS5 group (p<0.05).

The effects of ACS on neuroendocrine hormones levels are shown in Figures 2C, 2D. At 50 d, CS5 group had a significantly higher level of T3 hormone than CS0 group and CS3 group (p<0.05). The levels of CORT in CS0 and CS3 groups were significantly higher than CS5 group (p<0.05). However, there were no significant differences in the levels of T3 and CORT between CS0 group and CS3 group (p>0.05). At Y12, T3 level of broilers was positively correlated with the duration of intermittent cold stimulation, and there was significant difference among three groups (p<0.05). The CORT level in CS0 group was significantly higher than that in CS3 and CS5 groups (p<0.05). Compared with the 50 d, only the CORT level in CS3 group decreased significantly (p<0.05). The level of T3 did not change significantly among the three groups between 50d and Y12 (p>0.05).

### Effects of intermittent cold stimulation on mRNA and protein expression levels of AMPK pathway genes

**Figure 3:**
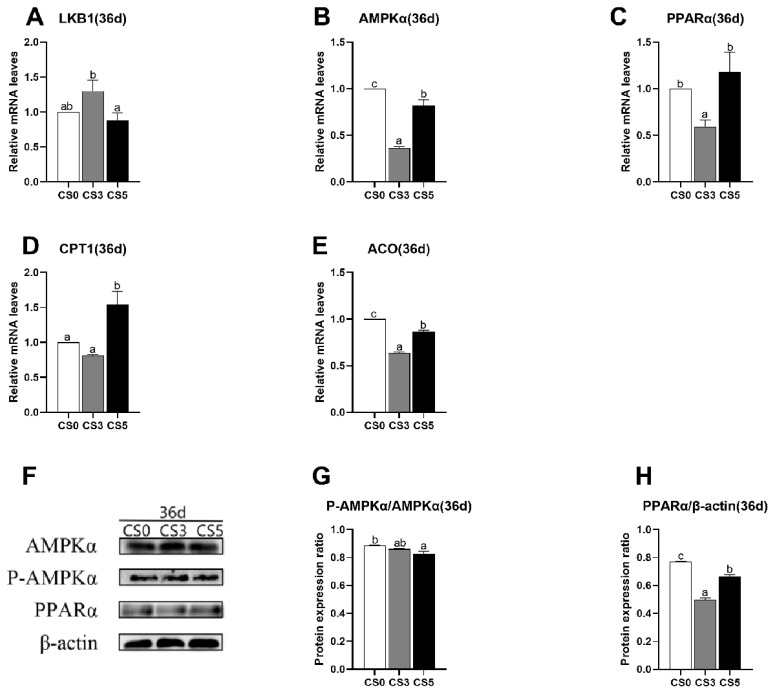
Effects of intermittent cold stimulation on mRNA and protein expression levels of AMPK pathway genes in broiler hearts.(A)–(E) Effect of intermittent cold stimulation on mRNA expression levels, (F)–(H) effect of intermittent cold stimulation on protein expression levels ^a–c^ The difference between the treatment groups was statistically significant (p<0.05). LKB1, Liver Kinase B1; AMPKα,adenosine monophosphate-activated protein kinase; PPARα, proliferators-activated receptor α; CPT1, carnitine palmitoyltransferase1; ACO, acyl -CoA oxidase; p-AMPKα, Phospho-AMPKα.

The effects of intermittent cold stimulation on mRNA expression levels of AMPKα pathway genes in the heart of broilers were shown in Figures 3A–3E. At 36 d, the AMPKα and ACO mRNA levels in CS0 group were extremely higher than CS3 and CS5 groups (p<0.05). The expression levels of AMPKα, PPARα, CPT1 and ACO mRNA in CS3 group were significantly lower than that in CS5 group (p<0.05), while the expression level of Liver Kinase B1 (LKB1) mRNA was significantly higher than that in CS5 group (p<0.05).

The effects of intermittent cold stimulation on protein expression levels of AMPK pathway genes were shown in Figures 3F–3H. At 36 d, the p-AMPKα/AMPKα ratio in CS0 group was significantly higher than that in CS5 group (p<0.05), while there was no significant difference compared with CS3 group (p<0.05). The expression level of PPARα protein in three groups significantly decreased first and then significantly increased with the duration of the intermittent cold stimulation, and there was significant difference among three groups (p<0.05).

### Effects of intermittent cold stimulation on mRNA and protein expression levels of mTOR pathway genes

**Figure 4:**
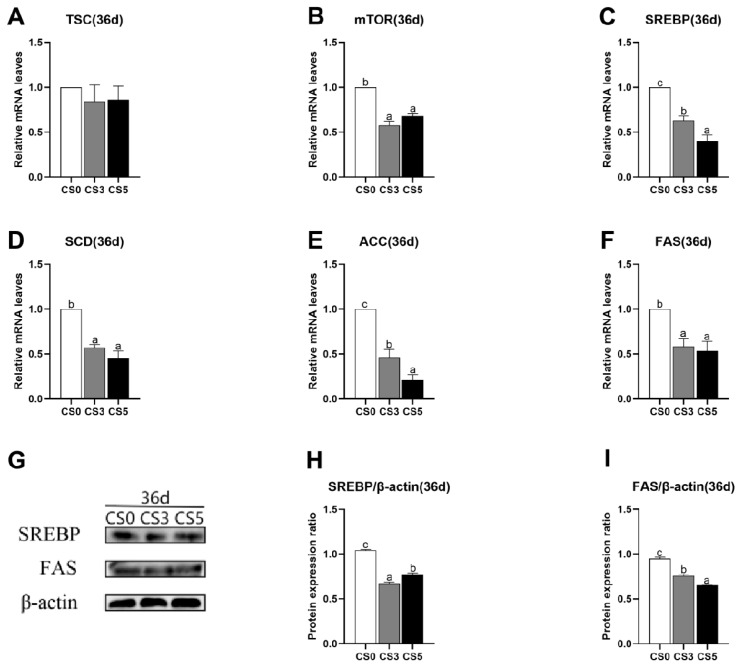
Effects of intermittent cold stimulation on mRNA and protein expression levels of mTOR pathway related genes in broiler hearts. (A)–(F) Effect of intermittent cold stimulation on mRNA expression levels, (G)–(I) effect of intermittent cold stimulation on protein expression levels. TSC, tuberous sclerosis complex; FAS, fatty acid synthase. ^a–c^ The difference between the treatment groups was statistically significant (p<0.05).

The effects of intermittent cold stimulation on mRNA expression levels of mTOR pathway related genes are shown in Figures 4A–4F. At 36d, mTOR, SREBP, SCD, ACC and fatty acid synthase (FAS) mRNA expression levels in CS0 group were significantly higher than CS3 group and CS5 group (p<0.05), while mRNA expression levels of tuberous sclerosis complex (TSC) were not significantly different among three groups (p>0.05).

The effects of intermittent cold stimulation on protein expression ratio of mTOR pathway related genes are shown in Figures 4G–4I. At 36–d, the expression levels of SREBP and FAS protein in CS3 group and CS5 group were significantly lower than those in CS0 group (p<0.05). In CS3 group, the expression level of SREBP was significantly lower than that in CS5 group (p<0.05), while the expression level of FAS was significantly higher than that in CS5 group (p<0.05).

### Effects of acute cold stress on mRNA and protein expression levels of AMPK pathway genes

**Figure 5:**
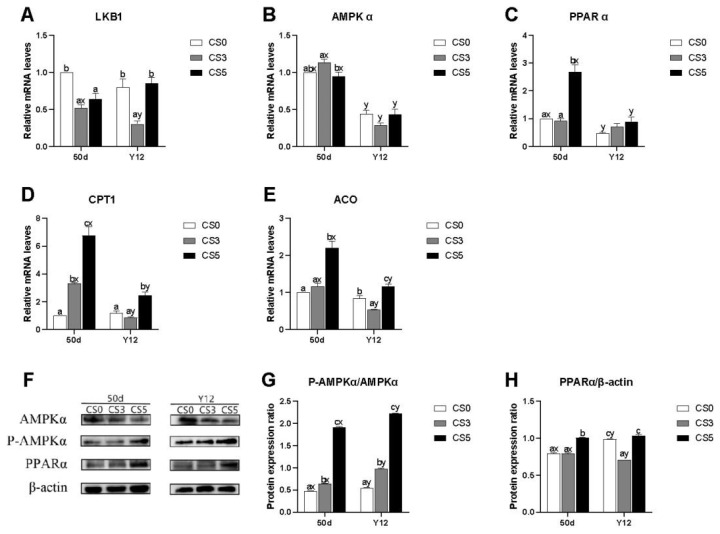
Effect of ACS on mRNA and protein expression levels of AMPK pathway related genes in broiler hearts. (A)–(E) Effect of ACS on mRNA expression levels. (F)–(H) effect of ACS on protein expression levels. ^a–c^ The difference between the treatment groups was statistically significant (p<0.05). ^x,y^ Represent the difference between the same treatment group at 50 d and Y12 (p<0.05). LKB1, Liver Kinase B1; AMPKα,adenosine monophosphate-activated protein kinase; PPARα, proliferators-activated receptor α; CPT1, carnitine palmitoyltransferase1;ACO, acyl-CoA oxidase; p-AMPKα, Phospho-AMPKα; ACS, acute cold stress

The effects of ACS on mRNA expression levels of AMPK pathway related genes were shown in Figures 5A–5E. At 50d, compared to CS0 group, the LKB1 mRNA expression levels in CS3 and CS5 group were significant decreased (p<0.05). The AMPKα mRNA expression level in CS0 group was not significantly different from that in CS3 and CS5 groups, but CS3 group had a significantly higher AMPKα mRNA expression level than that in CS5 group (p<0.05). The expression levels of PPARα, ACO and CPT1 mRNA in CS5 group were significantly higher than those in CS0 group and CS3 group (p<0.05). However, there were no significant differences in the expression of ACO and PPARα mRNA between CS3 group and CS0 group (p>0.05). At Y12, the expression level of LKB1 mRNA in CS3 group was significantly lower than that in CS0 group and CS5 group (p<0.05), while the expression levels of CPT1 mRNA were no significant differences between CS0 group and CS5 group (p>0.05). There were no significant differences in the mRNA expression levels of AMPKα and PPARα among the three groups (p>0.05). The expression levels of CPT1 and ACO mRNA in CS5 group were significantly higher than those in CS0 and CS3 groups (p<0.05). Compared with 50d, AMPKα and PPARα mRNA expression levels in CS0 group were significantly decreased (p<0.05). LKB1, AMPKα, CPT1 and ACO mRNA expression levels in CS3 group were significantly decreased (p<0.05). AMPKα, PPARα, CPT1 and ACO mRNA expression levels in CS5 group were significantly decreased (p<0.05).

The effects of ACS on protein expression levels of AMPK pathway related genes were shown in Figures 5F–5H. At 50 d, the expression of p-AMPKα/AMPKα was positively correlated with the duration of intermittent cold stimulation, and there was significant difference among three groups (p<0.05). The expression level of PPARα protein in CS5 group was significantly higher than that in CS0 and CS3 groups (p<0.05). At Y12, the expression of p-AMPKα/AMPKα in CS5 group was significantly higher than that in CS0 and CS3 groups (p<0.05). The protein expression of p-AMPKα /AMPKα in CS3 group was significantly higher than that in CS0 groups (p<0.05). The expression levels of PPARα in CS0 group and CS5 group were significantly higher than CS3 group (p>0.05). Compared with 50d, the expression of p-AMPKα/AMPKα was significantly increased among three groups (p<0.05). The protein expression of PPARα was significantly increased in CS0 group (p<0.05), significantly decreased in CS3 group (p<0.05), while no significant difference was observed in CS5 group (p>0.05).

### Effects of acute cold stress on mRNA and protein expression levels of mTOR pathway related genes

**Figure 6:**
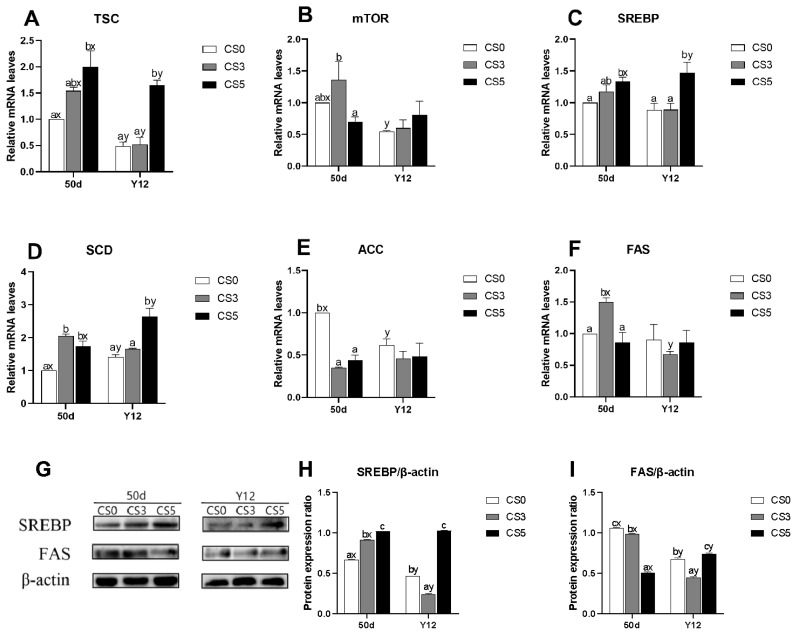
Effect of ACS on mRNA and protein expression levels of mTOR pathway related genes in broiler hearts. (A)–(F) Effect of ACS on mRNA expression levels. (G)–(I) Effect of ACS on protein expression levels. TSC, tuberous sclerosis complex; FAS, fatty acid synthase. ^a–c^ The difference between the treatment groups was statistically significant (p<0.05). ^x,y^ Represent the difference between the same treatment group at 50 d and Y12 (p<0.05). mTOR, mammalian target of rapamycin; SREBP, sterol-regulatory element binding protein; SCD, stearoyl-coA desaturase; ACC, acetyl-coA carboxylase; ACS, acute cold stress.

The effects of ACS on mRNA expression levels of mTOR pathway related genes were shown in Figures 6A–6F. At 50d, CS5 group had significantly higher TSC, SREBP and SCD mRNA expression levels than CS0 group (p<0.05), but there were no significantly differences between CS5 and CS3 groups (p<0.05). CS3 group had a significantly higher mTOR mRNA expression level than CS5 group (p<0.05), and a significantly higher FAS mRNA expression level than other groups (p<0.05). CS0 group had a significantly higher ACC mRNA expression level than CS3 group and CS5 group (p<0.05), but there were no significant differences between CS3 group and CS5 group (p<0.05). At Y12, CS5 group had significantly higher expression levels of TSC, SREBP and SCD mRNA than CS0 group and CS3 group (p<0.05). There were no significant differences in the expression levels of mTOR, ACC and FAS mRNA among three groups (p<0.05). Compared with 50d, the expression levels of TSC, mTOR and ACC mRNA in CS0 group were significantly decreased (p<0.05). The expression levels of TSC and FAS mRNA in the CS3 group were significantly decreased (p<0.05), while the expression levels of mTOR, SREBP, SCD and ACC mRNA had no significant difference (p>0.05). The expression levels of SREBP and SCD mRNA in CS5 group were significantly increased (p<0.05), while the expression levels of mTOR, ACC and FAS mRNA had no significant difference (p<0.05).

The effects of ACS on protein expression levels of mTOR pathway related genes were shown in Figures 6G–6I. At 50d, the protein expression of SREBP was positively correlated with the duration of intermittent cold stimulation among the three groups (p<0.05), while the expression level of FAS protein was negatively correlated with the duration of intermittent cold stimulation among the three groups (p<0.05). At Y12, the expression levels of SREBP and FAS protein in CS5 group were significantly higher than CS0 group (p<0.05), while those in CS3 group were significantly lower than CS0 group (p<0.05). Compared with 50d, the expression levels of SREBP and FAS protein in CS0 and CS3 groups were significantly decreased (p<0.05), while the expression level of SREBP protein in CS5 group had no significant difference (p<0.05) and the expression level of FAS protein in the CS5 group was significantly increased (p<0.05).

## DISCUSSION

The heart requires a significant amount of energy to maintain its contractile activity. Fatty acids are the preferred substrate for energy metabolism in the heart, with over 70% of energy derived from them [17]. As an organ with high lipid metabolic rate, the heart needs to maintain a high energy conversion efficiency for effective contraction in a low temperature environment [18]. Therefore, heart lipid metabolism is one of the important factors in adapting to cold environments and meeting specific energy demands. However, it remains unclear whether intermittent cold stimulation directly impacts broilers heart lipid metabolism and subsequently their adaptability to ACS.

Evidence suggests that the activity of hormones and molecules related to energy metabolism undergoes seasonal or temperature-induced changes. These processes are crucial for maintaining body temperature and generating heat in warm-blooded animals in cold temperature [19]. T3 is the active metabolite of thyroid hormone. Research indicates that in cold environments, T3 can significantly upregulate the expression of Uncoupling protein 1 (UCP1), leading to the uncoupling of ATP synthesis from oxidative phosphorylation, resulting in the release of energy in the form of heat. Therefore, the increase in T3 concentration in cold environments is believed to contribute to improved cold tolerance in animals [20]. CORT, the primary glucocorticoid in birds, increases under stress conditions to mobilize energy resources for survival, but prolonged exposure to high levels can negatively affect cellular immunity and production performance in broilers [11,21]. In present study, at 36d, both T3 and CORT concentrations in the CS3 group were significantly lower than in the CS0 group, while there were no significant differences in these indicators between the CS5 group and the CS0 group. This suggests that the intermittent cold stimulation protocol is mild and does not activate the HPT axis and HPA axis in broilers. Additionally, research by Wen et al. demonstrates that cold acclimation at 5°C significantly increases serum T3 level in striped hamsters and significantly enhances mitochondrial state-4 respiration and lipid breakdown capacity to maintain body temperature, providing further evidence for the involvement of T3 hormone in the cold acclimation process [22]. At Y12, the T3 levels in the CS3 group and CS5 group were significantly higher than that in the CS0 group. Therefore, we believe that broilers subjected to intermittent cold stimulation in the early stage can quickly activate the HPT axis under ACS conditions to regulate internal energy allocation and adapt to ACS. Ge et al. found that mice exposed to 4°C for 5 hours exhibited significantly higher plasma CORT levels compared to mice kept at room temperature (25°C), accompanied by an increase in the organism's anti-inflammatory levels. This to some extent enhanced the cold adaptability of the mice [23]. However, in a study by Hu, a high level of CORT can induce stress responses in broilers [24]. In present study, at Y12, the CORT levels in the CS3 group and CS5 group were significantly lower than that in the CS0 group. This may be because broilers in the CS3 and CS5 groups may have already adapted to the cold, resulting in a lower degree of activation of the HPA axis and thus not eliciting intense stress responses.

AMPK, a phylogenetically conserved serine/threonine protein kinase, is gaining attention as a crucial pathway for intracellular energy regulation. Its activation relies on the upstream phosphorylation of the α subunit at Thr-172 by LKB1. AMPK activation in the heart leads to downstream changes in gene expression, regulating fatty acid oxidation, glucose transport and protein metabolism, thereby increasing ATP production [25]. PPARα, a downstream gene of AMPKα, maintains cellular energy homeostasis by enhancing the expression of key genes involved in β-oxidation [26]. Experimental evidence demonstrates that mice with chronic AMPK activation exhibit high fatty acid oxidation capacity, leading to decreased triglyceride (TG) levels in the liver [27]. In the study by Wu, crayfish subjected to 9°C cold stress showed significant upregulation of AMPKα gene expression in the hepatopancreas, accompanied by downregulation of lipogenesis gene expression [28]. Furthermore, increased phosphorylation levels of AMPKα can mediate mitochondrial homeostasis, promote the function of CPT1 in fatty acid transport, and alleviate energy stress induced by low temperatures in pigs [29]. In the present study, contrary to previous research results, at 36d, mRNA levels of AMPKα and ACO, as well as the protein level of PPARα in the hearts of broilers in the CS3 and CS5 groups, were significantly reduced compared to those in the CS0 group. This indicates that the AMPK pathway in the hearts of broilers in the CS3 and CS5 groups was not activated, suggesting that intermittent mild cold stimulation does not affect the lipid decomposition capacity of broilers hearts. However, at Y12, compared to the CS0 and CS3 groups, mRNA levels of CPT1 and ACO, as well as the p-AMPKα/AMPKα protein level, were significantly upregulated in the CS5 group. In summary, compared to the CS0 and CS3 groups, the CS5 group is capable of decomposing more lipids for energy supply during ACS, thus preventing lipid metabolism disorders in the heart and maintaining normal function.

It is generally believed that the activation of AMPKα can stimulate TSC, thereby inhibiting the expression of mTOR and suppressing the synthesis of intracellular fatty acids [30]. As a downstream molecule of mTOR, SREBP maintains lipid metabolic homeostasis by positively regulating FAS and ACC [31]. ACC catalyzes the first step in FAS, converting acetyl-CoA to malonyl-CoA, while FAS synthesizes long-chain fatty acids from acetyl-CoA and malonyl-CoA. Therefore, the levels and activity of ACC and FAS determine the capacity for FAS [32,33]. Wang's research found that sustained activation of AMPKα in cold environments attenuates hepatic cell autophagy and apoptosis mediated by the PI3K/mTOR pathway, thereby enhancing the ability of fish to cope with cold stress [34]. Moreover, studies have shown that mTOR knockout in mice leads to disruptions in heart lipid metabolism and dysfunction [35]. Additionally, cold acclimation inhibits the activity of ACC and FAS in the liver of animals, enabling the liver to maintain its capacity to regulate lipid metabolism under high energy demand [32]. At 36d, except for TSC, all other mTOR pathway genes in the CS3 and CS5 groups were significantly lower than those in the CS0 group. This indicates that the intermittent cold stimulation can effectively induce adaptive changes in lipid synthesis metabolism in the hearts of broilers, thereby maintaining the energy homeostasis of broilers to adapt to cold stress. However, at Y12, compared to the 50d, the mRNA levels of mTOR and ACC were significantly downregulated in the CS0 group, while the mRNA and protein levels of FAS were significantly downregulated in the CS3 group. This indicates that the synthesis metabolism of heart fat in both CS0 and CS3 groups of broilers was inhibited by ACS.

Research has shown that the SREBP-SCD axis regulates the biosynthesis of monounsaturated fatty acids, thereby modulating the fluidity of cell membranes to maintain critical biophysical properties [36]. Additionally, the expression of the SCD gene is closely associated with low temperatures and plays a significant role in enhancing the adaptability of organisms [37]. In present study, at Y12, the mRNA expression level of SCD in the CS5 group was significantly higher than CS0 and CS3 groups, and significantly increased compared to 50d. This suggests that broilers subjected to intermittent cold stimulation can effectively mobilize the SREBP-SCD axis in the heart during ACS to enhance cell membrane fluidity and resist cold stress damage.

It is worth noting that studies have shown that hormones from both the HPA and HPT axes can activate the AMPK/mTOR pathway [20,38,39]. Research has revealed that CORT upregulates the gene expression of the AMPKα and mTOR in broilers [24]. Additionally, T3 enhances β-oxidation in mice primary brown adipocytes while diminishing mTOR activity [8]. However, the potential link between cold stress, neuroendocrine responses and heart lipid metabolism requires further investigation. In this study, at Y12, both the CS3 and CS5 groups exhibited significantly higher levels of T3 hormone and p-AMPKα/AMPKα compared to the CS0 group, while the CORT levels in CS3 and CS5 groups were significantly lower than in the CS0 group. Therefore, we speculate that it may be T3 rather than CORT that mediates the changes in gene expression of the AMPK pathway in the hearts of broilers under cold stress, thereby affecting heart fatty acid oxidation capacity. However, the specific regulatory mechanisms involved require further investigation.

## CONCLUSION

The findings of present study showed that intermittent cold stimulation at 3°C lower than the normal rearing temperature for 5 h can regulate hormone levels of the HPT and HPA axis, activate AMPK pathway and inhibit the mTOR pathway to maintain their heart lipid metabolism homeostasis. However, the above regulating effects were not significant with intermittent cold stimulation for 3h. Thus, intermittent cold stimulation at 3°C lower than the normal rearing temperature for 5h could help broilers adapt to the cold temperature environment by promoting heart lipid metabolism.

## Figures and Tables

**Table 1:** qPCR primers sequence

Genes	Serial number	Primer sequence (5’ - 3’)
*AMPKα*	NM_001039603.1	Forward: CGGAGATAAACAGAAGCACGAG Reverse: CGATTCAGGATCTTCACTGCAAC
*SREBP*	AY029224	Forward: GCCCTCTGTGCCTTTGTCTTC Reverse: ACTCAGCCATGATGCTTCTTCC
*PPARα*	NM_001001464	Forward: CAAACCAACCATCCTGACGAT Reverse: GGAGGTCAGCCATTTTTTGGA
*ACO*	NM_001185039	Forward: GATTTTTTGCAGGCGGGTATT Reverse: CACACGCTGGTTCACCTGAGT
*ACC*	NM_205505	Forward: GCTGGGTTGAGCGACTAATG Reverse: GGGAAACTGGCAAAGGACTG
*FAS*	NM_205155	Forward: TGAAGGACCTTATCGCATTGC Reverse: GCATGGGAAGCATTTTGTTGT
*SCD*	NM_204890.2	Forward: CAAGTTCTCCGAGACGCATG Reverse: GGGCTTGTAGTATCTCCGCT
*CPT1*	NM_001012898	Forward: GCCCTGATGCCTTCATTCAA Reverse: ATTTTCCCATGTCTCGGTAGTGA
*LKB1*	NM_001045833.1	Forward: GGACAGGTGCCTGAGGAGGAG Reverse: GGTGGAGAGCTTGCGGATCTTG
*TSC1*	NC_006104.5	Forward:TCCCACTGACTGTAGGTTCACTCC Reverse:CATGCTCATCACACTGGCTCTCAC
*mTOR*	NC_006108.5	Forward: GAAGTCCTGCGCGAGCATAAG Reverse: TTTGTGTCCATCAGCCTCCAGT
*β-actin*	NM_205518.1	Forward: CACCACAGCCGAGAGAGAAAT Reverse: TGACCATCAGGGAGTTCATAGC

qPCR, quantitative polymerase chain reaction.

**Table 2:** The dilution ratio of antibodies used in the current study

Antibody name	Dilution ratio
AMPKα (Wanleibio, Shenyang, China)	1:1,000
p-AMPKα (Wanleibio, Shenyang, China)	1:500
PPARα (Wanleibio, Shenyang, China)	1:1,000
SREBP (Wanleibio, Shenyang, China)	1:1,000
FAS (Wanleibio, Shenyang, China)	1:500
T β-actin (Zenbio, Chengdu, China)	1:9,000
